# Design and Heterologous Production of Bagworm Silk-Inspired Proteins in *Aspergillus oryzae*

**DOI:** 10.34133/csbj.0233

**Published:** 2026-09-30

**Authors:** Adiphol Dilokpimol, Ulrike Abendroth, Mari Nyyssönen, Anna S. Borisova, Anni Seisto, Jenni Muli, Dominik Mojzita, Fiona Småros, Hans Mattila, Antti P. Aalto, Mari Valkonen, Pezhman Mohammadi

**Affiliations:** VTT Technical Research Center of Finland, 02150 Espoo, Finland.

## Abstract

Silk-inspired proteins are attractive biomacromolecules owing to their structural diversity, sustainability, and tunable physicochemical properties. Among natural silks, the bagworm silk produced by *Eumeta variegata* has attracted increasing attention because of its unique sequence architecture and remarkable reported mechanical performance. However, the recombinant production of bagworm silk-inspired proteins remains largely unexplored. In this study, we designed and heterologously expressed 2 bagworm silk-inspired protein variants in *Aspergillus oryzae* using a synthetic expression system. The variants comprised a representative bagworm silk with an alanine- and glycine-rich repeat motif flanked by carbohydrate-binding modules at the N- and C-termini. Expression screening in engineered *A. oryzae* strains demonstrated stable accumulation of C-tag-reactive species corresponding to both variants in a protease-deficient host, whereas comparable accumulation was not detected in a carbon-catabolite-repressor-deficient strain under the tested conditions. The selected variant was further produced in a 2-l bioreactor, demonstrating the feasibility of scale-up and establishing *A. oryzae* as a promising platform for bagworm silk-inspired protein production. The recovered H-eBGW-containing protein fractions were readily processed into thin films and exhibited temperature-dependent changes in mechanical properties and surface wettability, demonstrating the processability and curing-responsive behavior of the recovered material. Collectively, our results demonstrate the feasibility of producing structurally complex bagworm silk-inspired proteins in filamentous fungi and provide insights into the design, expression, and processing of recombinant silk-inspired proteins for future bio-based applications.

## Introduction

Polymeric materials derived from petrochemicals, e.g., polyethylene, polypropylene, nylon, and polyester, are used in various industries due to their durability, flexibility, lightweight nature, and ease of processing, which enable diverse designs and cost-effective solutions [1–7]. However, their resistance to aging and biodegradation leads to persistent environmental burden after disposal. Bio-based materials derived from renewable resources offer a sustainable alternative and are increasingly used in applications ranging from packaging and textiles to construction [8–11]. Among biopolymers, protein-based materials have begun to emerge as particularly promising resources. They are assembled from amino acids and can be produced by biotechnology. Recombinant protein materials can be produced through biotechnological routes using renewable feedstocks and can provide molecular functionalities that are difficult to obtain with conventional petrochemical polymers; their overall environmental performance, however, depends on the production and downstream-processing scheme [12,13]. Natural protein-based materials such as silk, keratin, collagen, elastin, and resilin provide structure, flexibility, and protection in biological systems, each offering distinct properties for material design [14–23]. A defining feature of protein biopolymers is their structural versatility through variations in amino acid sequence, molecular weight, and supramolecular organization. A vast array of material behaviors can be designed from the molecular level, ranging from flexibility and extensibility to remarkable strength and resilience. Combined with biocompatibility and biodegradability, this molecular tunability positions protein-based materials at the forefront of next-generation sustainable solutions for biomedicine, tissue engineering, and advanced materials [1,13,23–25].

Silk is an iconic material in the textile industry due to its exceptional mechanical attributes, including remarkable tensile strength, impressive toughness, and elasticity [26–28]. It is a natural protein fiber composed of 2 main components: fibroin and sericin. Fibroin forms the threadlike core of the silkworm silk fiber (70% to 80%), while sericin (20% to 30%) acts as a glue to encapsulate and protect fibroin fibers [29–33]. While both fibroin and sericin contribute to the silkworm silk structure, fibroin constitutes the main structural phase and is the component most widely studied in biomaterials, where its processing-dependent molecular organization is associated with strong mechanical performance, favorable biocompatibility, and tunable degradation behavior. These properties make it a valuable biomaterial for tissue engineering, drug delivery, and wound healing [34–36]. Fibroins can have molecular weights ranging from dozens to hundreds of kilodaltons. The primary structure of fibroin contains highly repetitive sequences of amino acids as Gly-X motifs, where X typically is alanine (A) and serine (S), arranged in a pattern that enables the formation of tightly packed β-sheets. These β-sheets are the foundation of silkworm silk’s remarkable mechanical properties [34,37,38].

The heterologous production of recombinant silk proteins and their variants represents one of the most promising routes toward the sustainable production of bio-based materials. The expression of recombinant silk proteins has been attempted in a range of expression host systems [39–49]. However, the limitations hindering industrial manufacturing primarily stem from low production yields, in most cases lower than 1 g/l depending on the type of silk proteins [1], which make it challenging to reach the economic feasibility of large-scale production and process robustness. Using filamentous fungi capable of secreting recombinant silk protein could help to overcome this challenge.

Apart from the domestic silkworm (*Bombyx mori*) and various spiders, certain species of terrestrial and aquatic arthropods, e.g., bees, ants, hornets, and caddis flies, produce silk at a certain stage in their life cycle, in particular during the transformation of larvae into a pupal stage [26,38]. Some arthropod silks also exhibit a unique combination of durability, exceptional tensile strength, and remarkable extensibility, surpassing the performance of other high-performance synthetic fibers [50–52]. Bagworm moths belong to the family Psychidae in the Lepidoptera order. The caterpillar larvae of bagworms weave the silk in combination with environmental materials like twigs and leaves to create a tough protective case for camouflage and defense from predators [53,54]. The mechanical properties and sequences of the silk from Japan’s largest and heaviest bagworm (*Eumeta variegata*) have been recently reported [55]. Bagworm silk possesses high tensile strength (2.0 GPa), extensibility (32%), and toughness (364.0 MJ/m^3^) and in most cases surpasses other silk types, including spider silk, making it exceptionally durable compared to other natural silks. The extraordinary strength and toughness of bagworm silk stem from the unique relationship between primary sequence and highly ordered hierarchical structure rarely seen in other silks [55]. The sequence motifs of bagworm silk comprise ~160 amino acid units, which are longer than those of most reported insect silks [55–57]. In addition, a nonpolyalanine sequence combines the characteristics of long Gly-X dipeptide sequences from *B. mori* silkworm silk and the Gly-Gly-Z tripeptide sequences (Z is mainly Ala or Tyr) from Saturniidae silkworm and spider dragline silks [55,58]. The polyalanine sequence in bagworm silk is unique with an extended sequence of over 20 Ala residues, whereas it is much shorter in other silks [55,57]. In this study, we heterologously produced 2 different variants of single motifs inspired by bagworm silk protein, aiming to assess their production feasibility by *Aspergillus oryzae* and evaluate the mechanical properties of the produced bagworm silk-inspired-protein-containing fractions. *A. oryzae* was chosen as a trial host because of its protein secretion capacity and the ease of genetic manipulation in laboratory. The first variant was derived from a motif from the heavy-chain fibroin protein of the bagworm moth *E. variegata* [55,59], and the second was an engineered variant of the glycine-rich region with GGYGG units, a motif inspired by the glycine-rich repeat sequences commonly found in spider dragline silk (e.g., major ampullate spidroin 1/major ampullate spidroin 2 [MaSp1/MaSp2]) [60]. Two *A. oryzae* strains were tested as heterologous hosts, a carbon-catabolite-repressor-deficient (∆*creA*) strain and a protease-deficient strain, and bagworm silk-inspired protein was detected from the latter one. The mechanical properties of films prepared from H-eBGW-containing enriched fractions were further analyzed. The bagworm silk-inspired protein (H-eBGW)-enriched fractions showed the ability to form films as a functional coating in which the surface wetting behavior was adjustable by a simple posttreatment step. This study is a showcase for producing alternative protein-based materials using a fungal host. The recombinant bagworm silk-inspired protein is a promising source for future development of innovative natural bio-based materials.

## Results and Discussion

### Design of silk variants

The tandem repeat motifs of bagworm heavy-chain silk fibroin are composed of a polyalanine block (>20 amino acids) and nonpolyalanine block (NPAB) sequences with a short nonrepetitive segment of ~5 to 8 residues inserted within the NPAB sequence [55]. We selected bagworm silk as a target because its unusual combination of long polyalanine blocks and glycine-rich repeat regions is linked to exceptional reported mechanical performance, making it an especially attractive and challenging candidate for recombinant production for advanced material fabrication.

Five tandem repeat motifs of bagworm heavy-chain fibroin were reported earlier [59]; here we selected one motif (eBGW, National Center for Biotechnology Information [NCBI] Protein accession GBP83861.1, fibroin, partial, UniProt A0A4C1Z4W5 [55,59]) to validate the production feasibility of bagworm silk in *A. oryzae* (see Materials and Methods). This stretch represents a typical tandem repeat unit within the central repetitive core domain of the protein. It exhibits the characteristic hybrid motif architecture of bagworm silk fibroin: a long polyalanine block interrupted by a single glutamate residue (AAAAAAAAAEAAAAAAAAAAA), followed by glycine-alanine-rich alternating motifs (e.g., AGAGAGSG, GAGAGG, and GGAGG), a short hydrophobic/charged linker segment (GSTLIIVDEGG), and additional GA/GS/GG-containing repeats. This structural organization is expected to be responsible for the exceptional tensile strength and toughness of *Eumeta* bagworm silk. A second engineered variant (eBGW1) was constructed by modifying the glycine-rich region. To investigate the effect of altering the flexible linker/amphiphilic regions, glycine-rich motifs were replaced with 12 repeats of GGYGG units, a motif inspired by the glycine-rich repeat sequences commonly found in spider dragline silk (e.g., MaSp1/MaSp2) [60].

Based on 3-dimensional structure prediction by AlphaFold, the polyalanine sequence in the bagworm silk motif possibly forms a helical structure. In addition, the long Gly-X or Gly-Gly-X NPAB after the short nonrepetitive segment in eBGW could form a large spiral with a diameter of around 15 Å, while 12 repeats of GGYGG of eBGW1 form a long-disordered loop structure (Fig. 1). Apart from the predicted helixes and spirals, the main architectural features of silk motifs, however, possess both intrinsically disordered and amphiphilic structures. To facilitate expression and solubility with added functionality for enhanced surface adhesion, a carbohydrate-binding module family 1 (CBM1) domain and a linker from glycoside hydrolase family 5 from *A. oryzae* were fused to flank the N- and C-termini of this mid-block fragment. This design is based on our earlier work demonstrating that fusion of binding domains to repetitive silklike structural proteins improves expression levels, reduce aggregation and improve solubility, and targeted immobilization on various surfaces (Fig. 2 and Fig. S1) [61–64]. Moreover, both bagworm silk variants were expressed using a synthetic expression system (SES) [65], which is functional in a broad spectrum of fungal species without the need for host-dependent optimization, with either the amylase A pre-signal peptide from *A. oryzae* RIB40 (AmyA pre-ss, Joint Genome Institute [JGI] ID Aspor1|3751) or the hydrophobin pre-signal peptide from *Trichoderma reesei* (TrHFB pre-ss, JGI ID Trire2|73173). The expression cassettes were constructed with or without the N28 pro-signal peptide lipase 3 from *Rhizopus oryzae* (N28 pro-ss [66]), followed by a kex 2 protease cleavage site (K2) to release the mature targeted protein, and a C-terminal C-tag. Both eBGW and eBGW1 constructs were targeted to express in 2 different loci, amylase C (*amyC*) or dihydrodipicolinate synthase (*gaaC*).

**Fig. 1.**
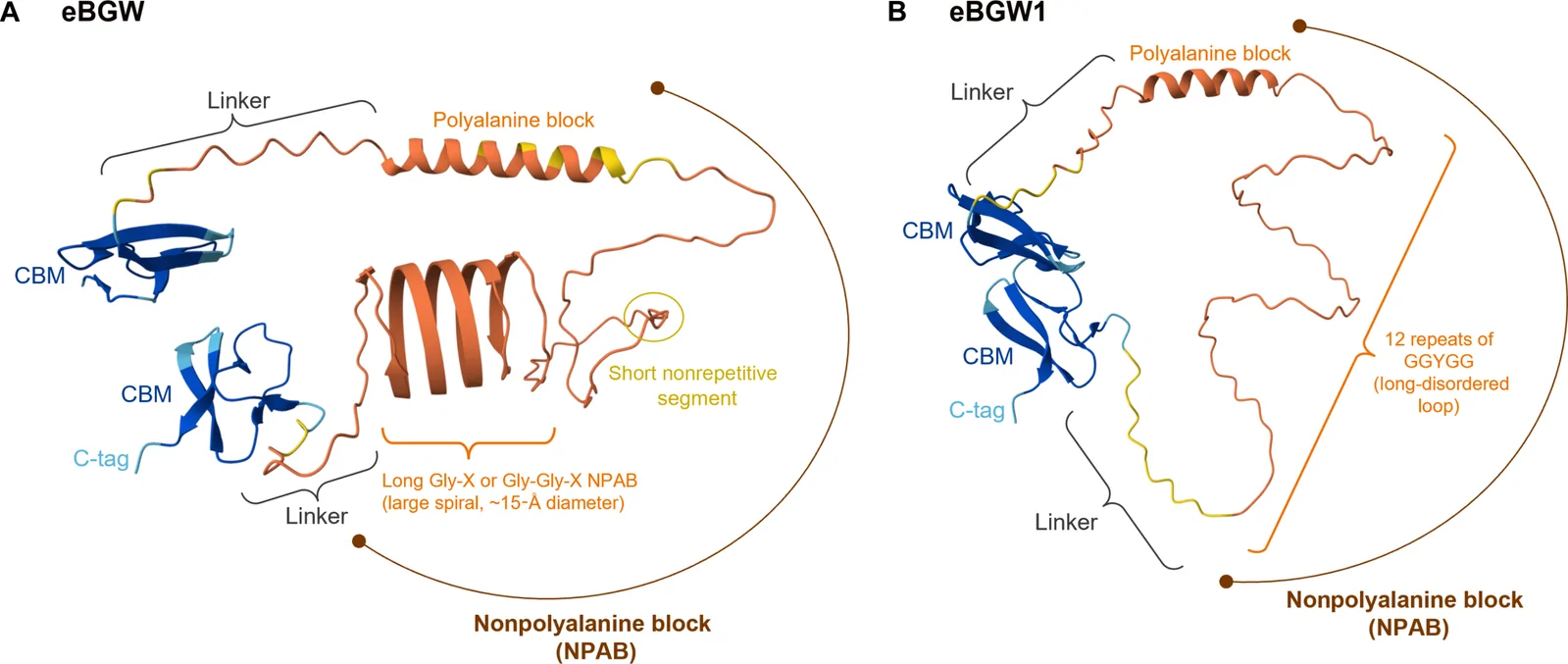
Three-dimensional (3D) structure models of 2 silk variants predicted by AlphaFold Server (AlphaFold3 [82]). (A) eBGW and (B) eBGW1 protein structure models including carbohydrate-binding modules (CBMs), linkers, polyalanine block, nonpolyalanine block (NPAB), short nonrepetitive segment, and a C-tag. Blue indicates a high predicted local distance difference test (pLDDT) score, while yellow and orange indicate low pLDDT scores. pLDDT was calculated by AlphaFold to estimate the accuracy of a predicted protein’s local structure.

**Fig. 2.**
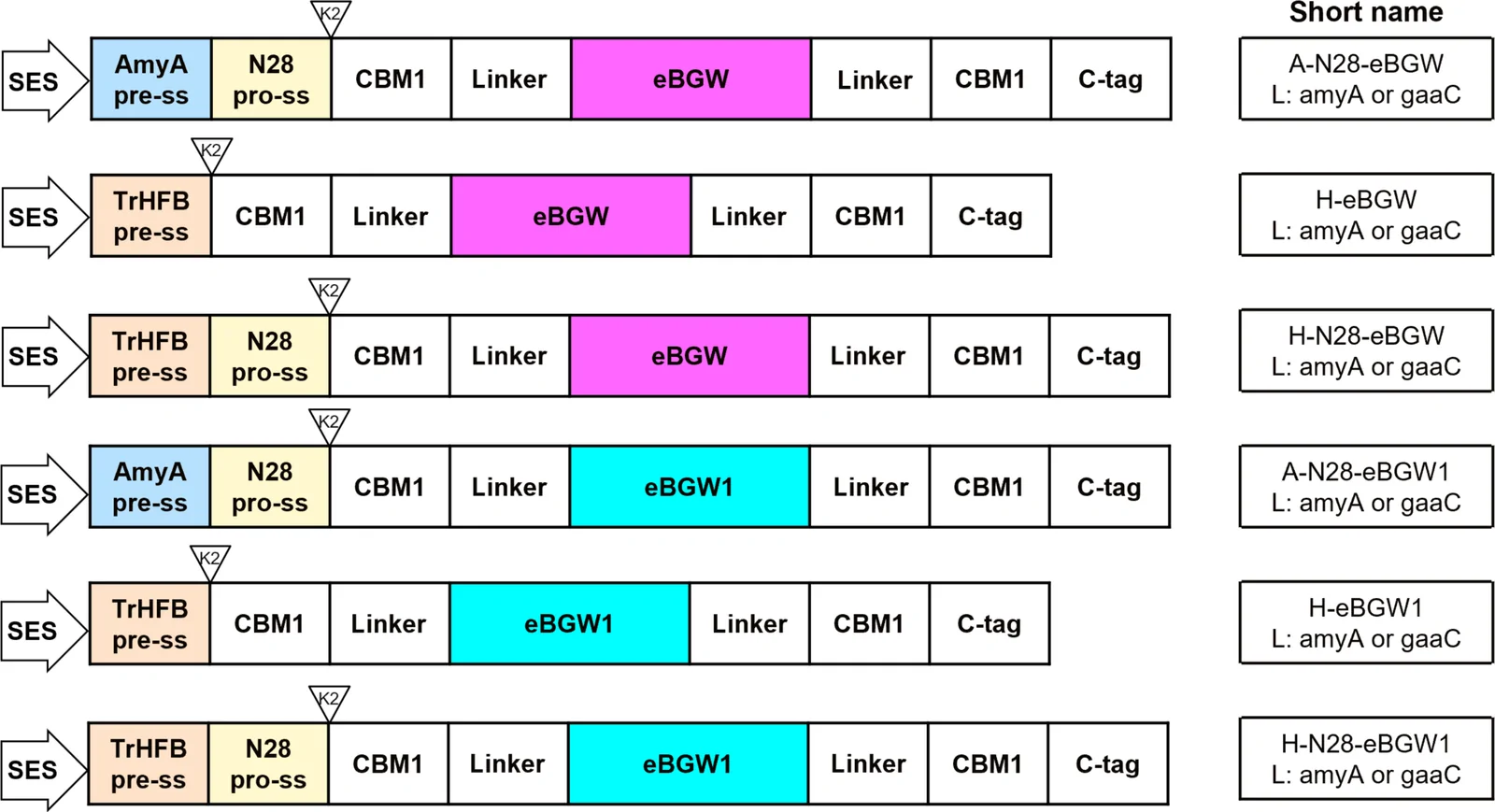
Overview of expression constructs of 2 bagworm silk variants. Two bagworm silk variants (eBGW and eBGW1) were expressed using a synthetic expression system (SES) with *Aspergillus* amylase A (AmyA) or *Trichoderma reesei* hydrophobin (TrHFB) pre-signal sequence (pre-ss) with or without the N28 pro-signal sequence (pro-ss) followed by a kex 2 cleavage site (K2). Carbohydrate-binding module 1 (CBM1) and its linker were conjugated on both the N- and C-termini of the silk variants. All constructs contain a C-tag at the C-terminus. The constructs target 2 different loci (L), amylase C (amyC), and dihydrodipicolinate synthase (gaaC).

### Expression of bagworm silk variants in a protease-deficient strain

The production of 2 bagworm silk variants was first tested in 2 modified *A. oryzae* strains: a protease-deficient (M5063) one and a carbon-catabolite-repressor-deficient (∆*creA*) one (Fig. 3). Earlier, it has been shown that the deletion of *creA/cre1* in aspergilli and *Trichoderma* led to higher production of many hydrolytic enzymes [67–70]. However, stable accumulation of intact C-tag-reactive eBGW/eBGW1 species was weak or not detected in the Δ*creA* strain under the tested cultivation conditions. In contrast, both variants were readily detected in the protease-deficient M5063 strain. Because detection was based on the C-terminal C-tag, the present assay does not distinguish between the degradation of the full recombinant protein and the proteolytic loss of the C-terminal region. Nevertheless, either mechanism would indicate limited stable accumulation of the intended intact recombinant product in the Δ*creA* background. We therefore continued the subsequent production studies with the protease-deficient M5063 strain.

**Fig. 3.**
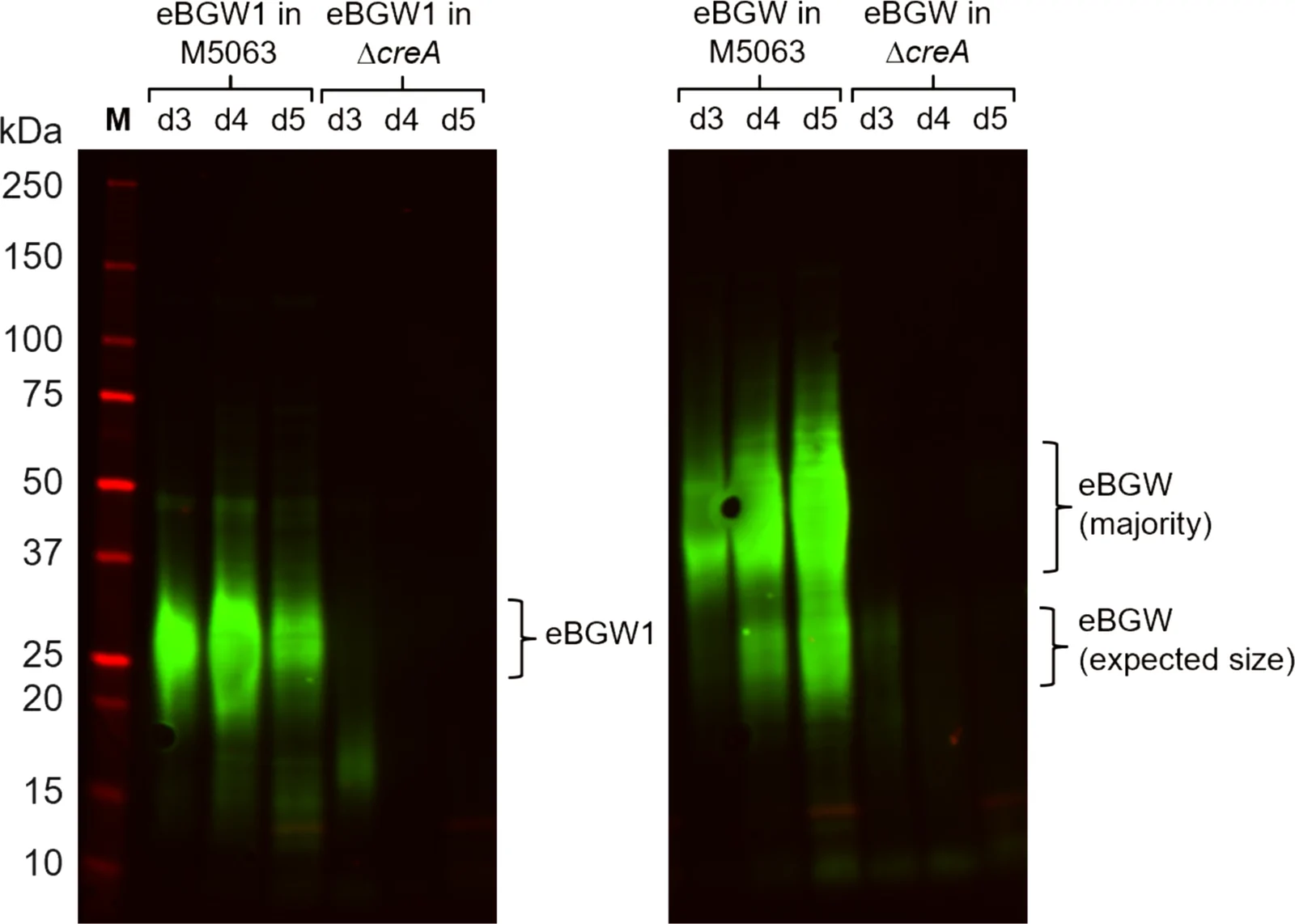
Detection of C-tag-reactive bagworm silk variants in 2 *Aspergillus oryzae* strains. Sodium dodecyl sulfate–polyacrylamide gel electrophoresis (SDS-PAGE) analysis of bagworm silk variant production from 2 *A. oryzae* strains, i.e., the protease-deficient (M5063) and ∆*creA* strains. The culture broth (11.25 μl of the supernatant) from days 3 to 5 of cultivation (d3 to d5) in a 24-well plate was analyzed by Western blotting using anti-C-tag antibody. The calculated masses of eBGW and eBGW1 are 17.5 and 22 kDa, respectively. M, protein marker.

Different constructs of bagworm silk variants were transformed into the M5063 strain (Fig. S2). Various degrees of production and degradation in a 24-well cultivation indicated that there were still remaining proteases targeting the silk variants. Since the specific proteases that degrade the bagworm silk protein are not known, a mix of protease inhibitors, including chymostatin, pepstatin, and soybean trypsin inhibitor (see Materials and Methods), were added to the 24-well cultivation. This confirmed the production of all silk variants by Western blot analysis using an anti-C-tag antibody (Fig. 4 and Fig. S3). In general, the bagworm silk protein production of both variants was rather similar in most constructs, indicating that both loci, i.e., *amyC* and *gaaC*, could be used for the production of the target protein with an SES. In addition, both pre- and pro-signal sequences performed equally well for production of silk variants. The masses of both variants were larger than the predicted ones (22 and 17.5 kDa, respectively). Both supernatants were treated with endoglycosidase H to remove glycosylation; however, no size reduction was observed (Fig. S4). This indicates that the larger mass may not be caused by glycosylation, but possibly by other modifications or multimerization. However, the high concentration of background proteins and the heterogeneity of H-eBGW make it not possible to use analytical methods, e.g., native polyacrylamide gel electrophoresis (PAGE) analysis, mass spectrometry, or mass analysis by high-performance liquid chromatography, to confirm different species of H-eBGW.

**Fig. 4.**
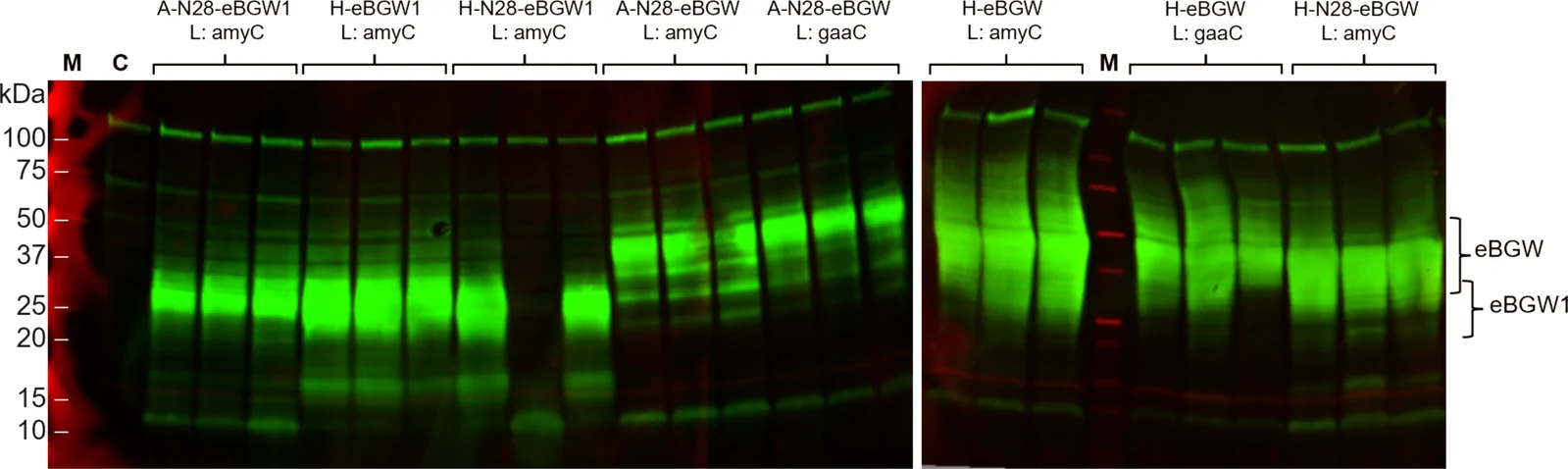
Production of 2 bagworm silk variants in an *Aspergillus oryzae* protease-deficient (M5063) strain with supplementation of protease inhibitors. Bagworm silk variant production (15 μl of supernatant) from different constructs was screened after 5 d of cultivation. The cultivation was done in a 24-well plate with buffered glucose-complex medium (BMGY) supplemented with a protease inhibitor mix of 10 μM chymostatin, 10 μM pepstatin, and 0.5 mg/ml soybean trypsin inhibitor (final concentration), from days 2 to 4. Western blot analysis using an anti-C-tag antibody. M, protein marker; C, *A. oryzae* protease-deficient strain without silk variant as a negative control.

### Production of a bagworm silk variant in a 2-l bioreactor

Based on the production level and low protein degradation, eBGW with the *T. reesei* hydrophobin pre-signal sequence (H-eBGW in M5063) was selected for test cultivation in a 2-l bioreactor to evaluate scalability and to test the mechanical properties of bagworm silk. The cell dry weight of the H-eBGW strain reached around 35 g/l on day 4, while that of the wild type reached around 30 g/l (Fig. S5). H-eBGW was produced in the bioreactor (Fig. 5); however, the supplementation of protease inhibitors was required to obtain intact protein, which limits current feasibility for large-scale production. To engineer a more suitable production strain, the specific proteases that degrade the silk protein can be identified and deleted, e.g., using a target-specific approach [71].

**Fig. 5.**
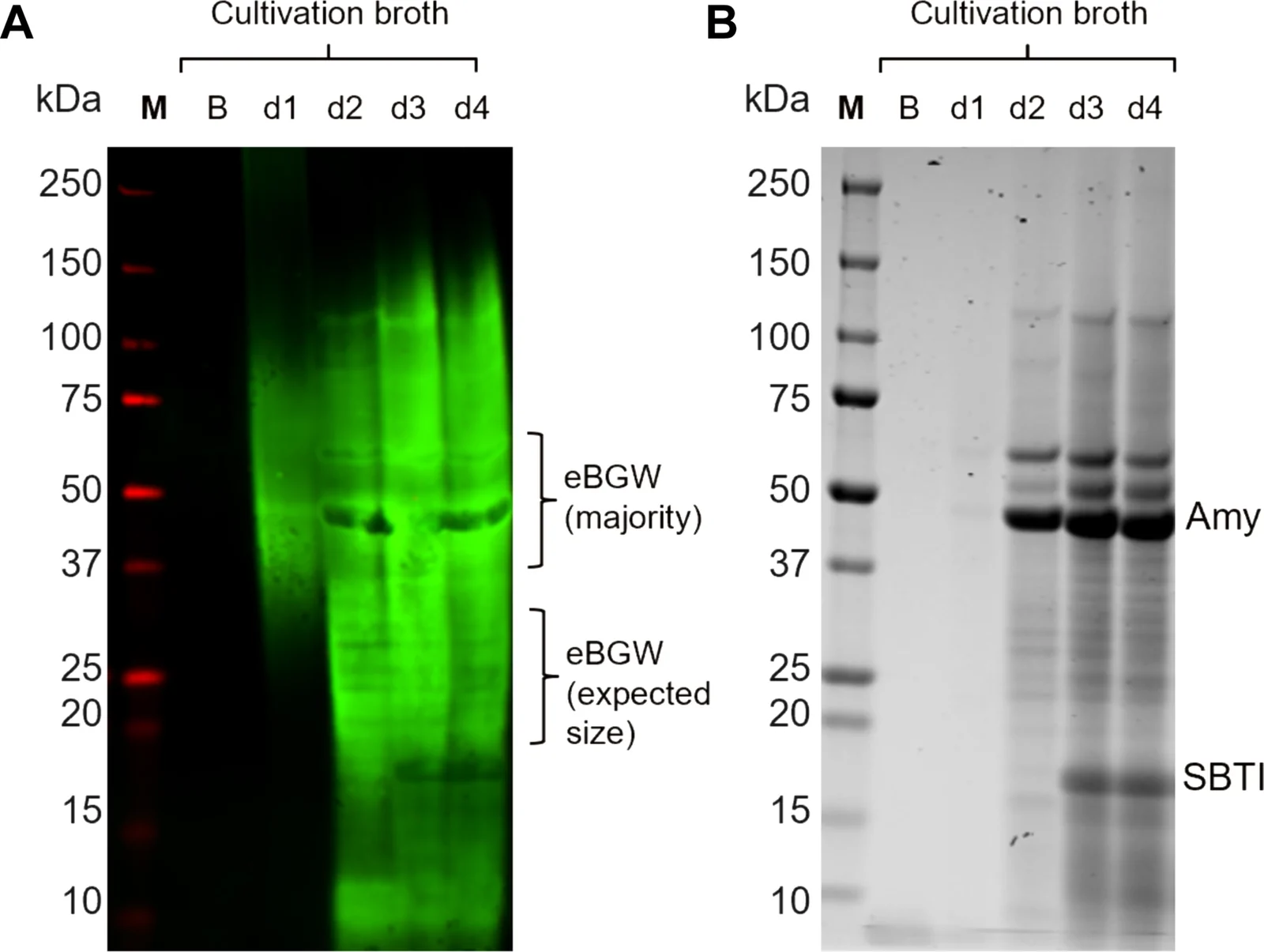
Production of H-eBGW in M5063 in a 2-l bioreactor. The culture broth (11.25 μl of the supernatant) from 1 to 4 d of cultivation was analyzed by sodium dodecyl sulfate–polyacrylamide gel electrophoresis (SDS-PAGE). The protease inhibitors, 10 μM chymostatin, 10 μM pepstatin, and 0.5 mg/ml soybean trypsin inhibitor (final concentration), were added on days 2 and 3. (A) Western blot analysis using an anti-C-tag antibody. (B) Coomassie blue-stained SDS-PAGE. M, marker; B, blank medium; d, days of cultivation. Amy indicates amylases, and SBTI indicates soybean trypsin inhibitor.

Sodium dodecyl sulfate (SDS)-PAGE analysis revealed a dominant protein band at approximately 50 kDa (Fig. 5B). In *A. oryzae*, α-amylase is among the most abundantly secreted proteins, and the RIB40 strain used in this study contains 3 highly similar α-amylase genes—*amyA*, *amyB*, and *amyC* (JGI IDs Aspor1|3751, Aspor1|4893, and Aspor1|8250, respectively), which share more than 95% sequence identity [72,73]). In the present work, expression was targeted only to the *amyC* locus; the remaining loci could therefore be exploited to introduce additional copies of the silk construct to further increase production levels. Removing background proteins from the genome has been shown to improve the production of the target protein in filamentous fungi [72]. In addition, reducing the amount of amylases in the culture supernatant would also likely substantially improve the downstream processing of the silk protein [74].

To harvest H-eBGW in the supernatant, the culture was filtered through XE50H and XE1200H filter sheets (see details in Materials and Methods) followed by a 0.45-μm polyethersulfone (PES) membrane to remove cell debris and possible contaminations. The filtered supernatant was then concentrated with buffer exchange to phosphate-buffered saline (PBS) pH 7.3 using tangential flow filtration with a 10-kDa molecular weight cutoff (MWCO; see Materials and Methods). After the filtration and concentration steps, 2 g of total protein (38 mg total protein/ml) was obtained. H-eBGW could not be clearly resolved as a discrete band by Coomassie-stained SDS-PAGE because of the background proteins in the culture supernatant. Consequently, H-eBGW production and its subsequent recovery were primarily monitored by anti-C-tag Western blotting, and its concentration could not be reliably estimated from the stained gel. As the mass of H-eBGW was larger than the calculated one (22 kDa) and the protein appeared smeary on the gel, the supernatant was treated with 8 M urea to solubilize the protein. The urea concentration was then reduced to 2 M, and the sample was treated with peptide:N-glycosidase F to remove glycosylation. While this resulted in the mass shift of many background proteins including amylases, no size reduction of H-eBGW was observed, confirming that the larger mass was not caused by glycosylation. In addition, we observed 2 major bands of 65 and 45 kDa from the Western blot analysis of the supernatant containing H-eBGW (Fig. S4c and d). These suggested that the bagworm silk-inspired protein might form an SDS-resistant multimer. As H-eBGW contains a C-terminal tag, affinity chromatography of the C-tagged protein was initially attempted. However, most of the silk protein did not bind to the column but remained in the flow-through (Fig. S6). Given the inefficient affinity capture observed here, retention of the tag is not necessarily required for future scale-up, and subsequent process development may employ either a tag-free construct or an alternative purification strategy better suited to the physicochemical behavior of H-eBGW. Since H-eBGW did not properly bind to the C-tag column, we instead attempted to purify this protein by size-exclusion chromatography. The concentrated supernatant was desalted and buffer exchanged to 50 mM MES pH 6.5 and applied twice to a prepacked HiLoad 26/60 Superdex 75 pg (see details in Materials and Methods). The supernatant was then purified twice by gel filtration with the aim of removing the major background bands (~50 to 75 kDa). However, after the second purification, the main amylases (~50 kDa) were still copurified with H-eBGW. Unexpectedly, after the gel filtration, the major bands of H-eBGW shifted lower to around 20 to 35 kDa, which is closer to the calculated mass (22 kDa). This could be either because of protease degradation or because the component that caused the multimerization was washed off during the downstream processing. Based on the SDS-PAGE analysis (Fig. 6), the fractions containing silk protein were pooled together (Fig. 6, pooled fractions), stored at −20 °C, and used for mechanical testing.

**Fig. 6.**
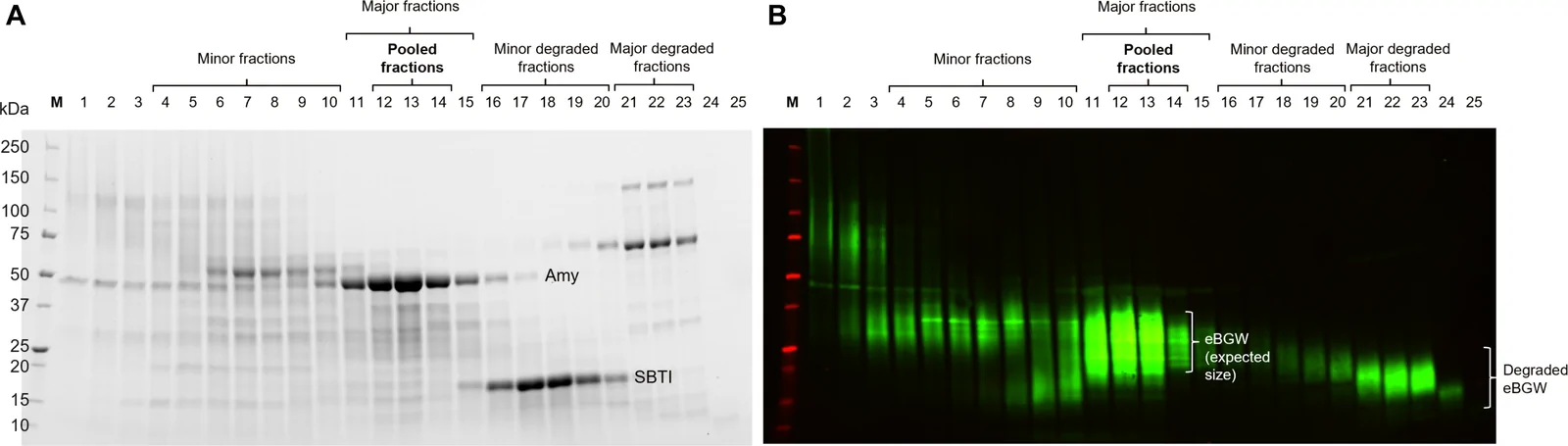
Purification of H-eBGW by size-exclusion chromatography. The filtered culture broth after harvesting was concentrated and buffer exchanged by ultrafiltration and subsequently purified by size-exclusion chromatography (PD MidiTrap G-25 and HiLoad 26/60 Superdex 75 pg). (A) Coomassie blue staining. (B) Western blot analysis using an anti-C-tag antibody. M, marker; numbers indicate purified fractions from HiLoad 26/60 Superdex 75 pg. “Pooled fractions” indicate the fractions that were collected for mechanical property analysis.

### High-performance film formation with process-controlled mechanical properties

Having established microbial expression and subsequent enrichment of H-eBGW-containing protein fractions, we next investigated whether the recovered material could be processed into coherent solid-state films. Because the films were prepared from an H-eBGW-containing enriched fraction rather than analytically purified H-eBGW, the following mechanical and surface measurements describe the properties of the recovered protein-containing material as a whole. As a first functional demonstration, the H-eBGW-containing enriched fraction was cast into thin films on silicon substrates, providing a scalable and reproducible platform to assess film formation quality and quantify mechanical performance. Based on Western blot analysis, pooled size-exclusion chromatography fractions containing H-eBGW (Fig. 6, pooled fractions) were used for film formation and functional testing. These experiments were intended to assess the processability and curing-responsive coating behavior of the recovered H-eBGW-containing fraction. Additionally, the film-on-substrate configuration also enabled direct comparison to our earlier silk-inspired coating studies, where recombinant silklike proteins were evaluated in thin-film architectures to link processing to mechanical reinforcement and interfacial performance [75,76]. The films were formed directly on silicon and subsequently posttreated at 22, 80, or 140 °C (Fig. 7A). Optical inspection indicated that films processed at 22 °C exhibited pronounced interference colors and spatial heterogeneity. Similar optical patterns in our previously reported silk-inspired coatings were associated with variations in film thickness and thin-film interference [75,76]. Accordingly, the heterogeneity observed here is consistent with local thickness variations and/or incomplete film leveling during drying. In contrast, films cured at 80 and 140 °C appeared more optically uniform at the micrometer scale, suggesting enhanced consolidation during thermal treatment (Fig. 7A). Similar visually apparent densification and property evolution upon thermal treatment were previously observed in our earlier silk-inspired coatings, where annealing was used as a practical route to strengthen the coating network [76]. To evaluate whether these processing steps also translate into measurable mechanical reinforcement in the H-eBGW-enriched fraction, we performed nanoindentation on H-eBGW-containing films. Load–depth curves showed a systematic reduction in penetration depth with increasing posttreatment temperature (Fig. 7B). At comparable maximum loads, 22 °C H-eBGW-containing films exhibited the deepest indents, whereas 80 and 140 °C films resisted deformation more strongly, indicating a stiffer and/or harder near-surface response after treatment. Consistently, the Ashby plot that illustrates the hardness–modulus map (Fig. 7C) revealed a monotonic increase in both hardness and modulus with increasing curing temperature. At 22 °C, the measurements were concentrated in the subgigapascal hardness range with moduli around 10 GPa. With increasing curing temperature, the distribution progressively shifted toward higher values, reaching approximately 1-GPa hardness and 50- to 60-GPa modulus at 140 °C. The magnitude of the increase demonstrates that the bagworm silk-inspired-protein-containing fractions can be processed into functional coatings with tunable mechanical performance for various technical applications. The observed strengthening trend is in line with our previous reports on spider silk-inspired coatings, where thermal posttreatments and protein-mediated film architectures enhanced coating robustness and load-bearing behavior [75,76]. It is worth mentioning that because the tested films were prepared from H-eBGW-containing enriched fractions rather than analytically pure single-component protein, the measured values should be interpreted as functional properties of the recovered coating material, not as intrinsic material constants of isolated H-eBGW.

**Fig. 7.**
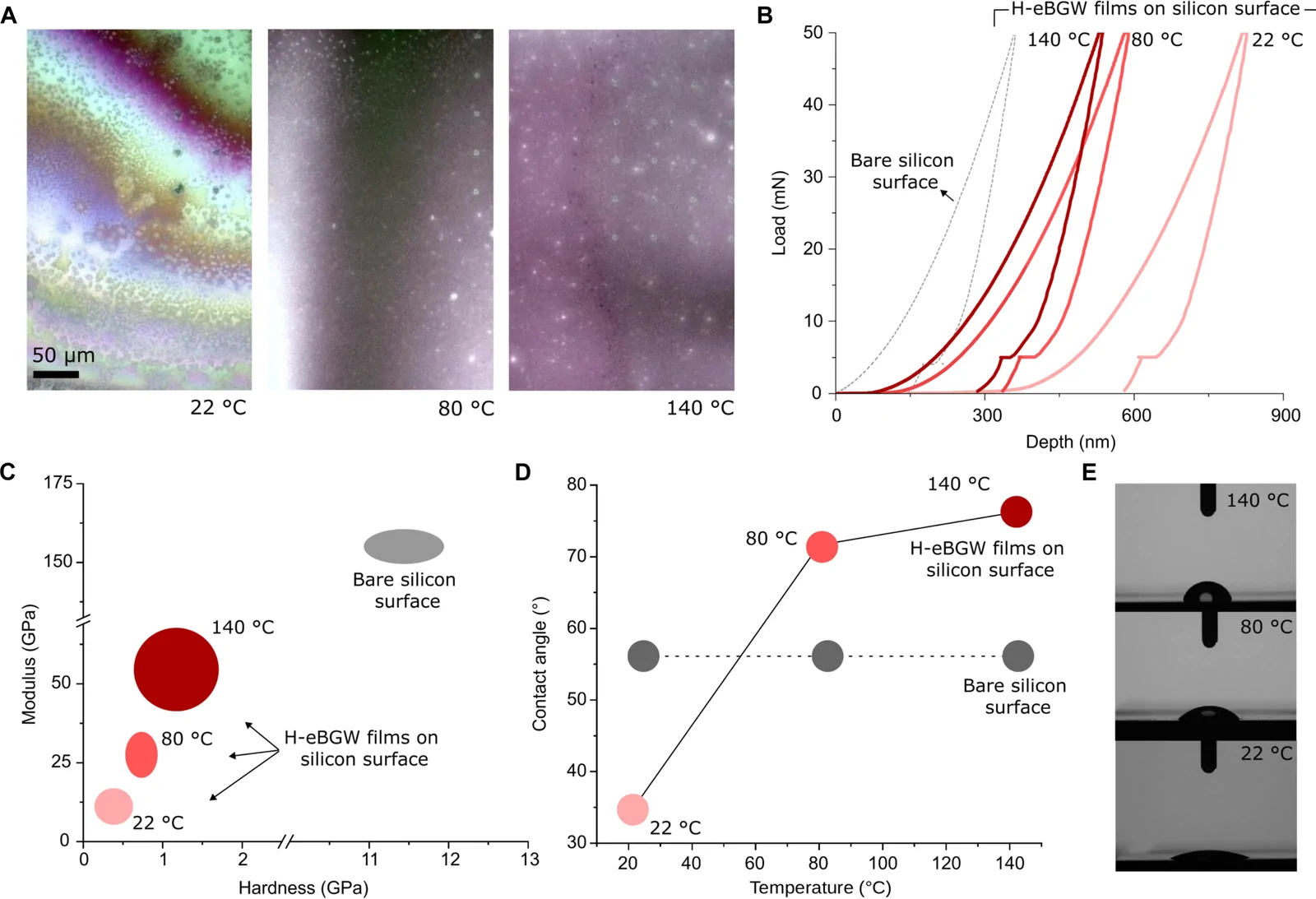
Film formation and tunable performance via thermal curing. (A) Optical images of H-eBGW-containing films formed on a silicon surface and cured at 22, 80, or 140 °C, showing a transition from strongly thickness-varied/interference-colored regions at 22 °C to more optically uniform films after thermal curing. Scale bar: 50 μm. (B) Representative nanoindentation load–depth curves for H-eBGW-containing films cured at 22, 80, and 140 °C (red tones). The bare silicon substrate response is shown for reference (gray, dashed). (C) Ashby-style hardness–modulus map obtained from nanoindentation measurements (*N* = 25) for H-eBGW-containing films cured at 22, 80, and 140 °C, together with the bare silicon control. Each circle/ellipse represents the distribution of measured hardness and modulus values for the corresponding condition; its horizontal and vertical dimensions indicate the spread of the measurements along the hardness and modulus axes, respectively. (D) Water contact angle of H-eBGW-containing films as a function of curing temperature compared to that of bare silicon (gray). (E) Representative side-view images of water droplets on H-eBGW-containing films cured at 22, 80, and 140 °C, consistent with the trend in (D).

Although this study does not directly examine detailed molecular structure or conformational changes, the observed curing-dependent trends are consistent with earlier reports on silklike systems, where posttreatment has been associated with network consolidation and altered intermolecular organization. In the present case, thermal curing may similarly promote densification of the engineered protein coating, including tighter molecular packing, stronger intermolecular interactions, and possible shifts in the balance between ordered and less ordered domains, together with the displacement of bound water. Such changes provide a plausible explanation for the increased resistance of the films to both elastic and plastic deformations during indentation.

### Thermal curing increases hydrophobicity, revealing a coupled mechanics–wettability trade-off

To assess whether the H-eBGW-containing films also offer tunable surface functionality, we then set out to quantify their wettability by water contact angle (WCA) measurements following postdeposition treatment. The results indicated a direct correlation: we noted an increase in contact angle with increasing curing temperature (Fig. 7D and E), from a relatively low value at 22 °C (~35°) to substantially higher values after thermal treatment at 80 °C (~70°) and 140 °C (~75°). In contrast, bare silicon remained approximately constant over the same temperature range (gray markers, Fig. 7D), indicating that the wettability changes are governed by the H-eBGW-containing film surface rather than by temperature-dependent changes in the substrate. Representative droplet images (Fig. 7E) mirrored this trend, with progressively more spherical droplets on films treated at higher temperatures. Notably, the increase in contact angle occurred in parallel with the increase in hardness and modulus (Fig. 7C and D), indicating a coupled evolution of mechanical reinforcement and surface wetting during posttreatment. A plausible interpretation is that thermal treatment alters both the near-surface network and the outermost surface chemistry/topography. Two nonexclusive mechanisms can explain the reduced wettability: (a) dehydration and densification of the protein network, which lowers the polar, water-accessible free volume and reduces the contribution of bound water, and/or (b) surface reconstruction via reorientation or segregation of side chains, enriching the interface in less polar (more hydrophobic) groups during annealing. This coupling between thermal processing, strengthening, and increased hydrophobicity is consistent with our earlier work on spider silk-inspired coatings, where annealing served as a practical lever to tune both mechanical properties and wettability [76]. More broadly, our prior interfacial-modulation study demonstrated that silk-inspired proteins can substantially influence coating performance at solid interfaces [75]; the bagworm silk-inspired-protein-containing coatings here extends that concept by showing that a microbially produced silklike protein can be processed into films whose surface wetting behavior is adjustable by a simple posttreatment step with potential use for advanced technical applications.

## Materials and Methods

### Background fungal strain construction and screening

Two *A. oryzae* strains, i.e., a protease-deficient strain (M5063) and a ∆*creA* strain (M6752), were used in this study. To create a basic production strain for both the protease-deficient and ∆*creA* strains, *A. oryzae* strain RIB 40 (NRRL 5590/ATCC 42149) was used as a starting background strain. First, the *ligD* and *pyrG* genes were deleted by cotransformation of *pyrG* and *ligD* deletion cassettes into *A. oryzae* RIB 40 to create a ∆*pyrG*∆*ligD*-based strain (Table 1; for details, see Fig. S1). The deletion of *pyrG* was to create a *pyrG*-based marker recycling system based on the uridine auxotrophic mutant and 5-fluoroorotic acid counterselection [77] and the deletion of *ligD* would enhance the homologous recombination [78]. Multiple construction steps were then performed to delete selected protease genes (*alpA*, *ocpB*, *cpyA*, *Np1*, and *tppA*) and a transcription factor gene (*ptrT*) to generate the protease-deficient strain (M5063). Synthetic transcription factor genes were also added during the engineering of M5063 to make it compatible with the SES-based expression of target genes [65] (Table; for details, see Fig. S1). The ∆*creA* strain (M6752) was generated by deletion of *creA* from the ∆*pyrG*∆*ligD*-based strain. In short, the deletion cassettes were constructed using 600- to 1,300-bp upstream and downstream DNA fragments of the target genes containing an overlap of the selection marker *pyrG* from *A. oryzae* (Fig. S1). The deletion cassette was transformed through protoplast-mediated transformation as previously described [79]. Deletion of target genes was performed using a *pyrG* marker recycling strategy based on Maruyama and Kitamoto [80] and Yoon *et al.* [81]. The deletions were confirmed by polymerase chain reaction (PCR) and/or quantitative PCR using primers toward the target genes (Table S1).

**Table 1.** List of the deleted genes and accession numbers used in this study

Gene name ^a^	JGI protein ID	AspGD accession	UniProt
*ligD*	8356	AO090120000322	Q2U6A1
*pyrG*	10736	AO090011000868	O13416
*creA*	5292	AO090026000464	Q9P889
*ptrT*	3752-3753	AO090003001211-2	A7BJS7
*alpA*	3604	AO090003001036	P12547
*ocpB*	7804	AO090701000220	Q2U908
*cpyA*	11066	AO090103000332	Q2TYA1
*Np1*	10032	AO090011000036	Q2U1G7
*tppA*	7179	AO090166000084	Q2U4G6

JGI, Joint Genome Institute; AspGD, Aspergillus Genome Database

^a^ Gene names were based on [83].

#### Bagworm silk-protein-producing strain construction and screening

Two variants of bagworm silk-protein motif sequence were used in this study, i.e.,

• eBGW: A​AAA​AAA​AAE​AAA​AAA​AAA​AAA​GGA​GGY​GPY​GGF​AGA​GAG​AGG​AGG​AGG​AGG​AGG​AG​STL​IIV​DE​GGY​GGA​GGA​GSG​AGS​GVG​AGA​GSG​AGA​GGA​GAA​AGA​GAG​AGS​GAG​S​GSG​AGA​GAG​SGA​GAG​SGA​GAG​AGA​GSG​AAGGAGAGAGAG (NCBI Protein accession GBP83861.1, fibroin, partial, UniProt A0A4C1Z4W5 [55,59])
• eBGW1: A​AAA​AAA​AAE​AAA​AAA​AAA​GGY​GGG​GYG​GGG​YGG​GGY​GGG​GYG​GGG​YGG​GGY​GGG​GYG​GGGYGGGGYGGGGYGGGGYGG (12 repeats of GGYGG), a motif inspired by the glycine-rich repeat sequences commonly found in spider dragline silk (e.g., MaSp1/MaSp2) [60]

The plasmids and primers used in this study are shown in Fig. 2, Fig. S1, and Table S1. CBM1 (GAVAHYYQCGGINYSGPTTCESGYTCVKQNPYYSQCL) and a linker (SGVVTATPTPSHPAPQP) from glycoside hydrolase family 5 from *A. oryzae* (JGI ID Aspor1|10597, Aspergillus Genome Database [AspGD] ID AO090011000715) were conjugated on both the N- and C-termini of the bagworm silk-protein motif sequence based on Borisova *et al.* [75]. Two different pre- and one pro-signal peptides, i.e., amylase A signal peptide from *A.* oryzae RIB40 (AmyA pre-ss, MMVAWWSLFLYGLQVAAPALA, JGI ID Aspor1|3751, AspGD ID AO090003001209), hydrophobin signal peptide from *T. reesei* (TrHFB pre-ss, MKFFAIAALFAAAAVAQP, JGI ID Trire2|73173), and N28 pro-signal peptide lipase 3 from *Rhizopus oryzae* (N28 pro-ss, VDDDNLVGGMTLDLPSDAPPISLSGSTNSA) [66], were used as secretion signals. The constructs under the SES [65] were synthesized and codon-optimized for expression in *A. oryzae* and cloned to a plasmid containing *amyC*- or *gaaC*-upstream and -downstream fragments by GenScript (Rijswijk, the Netherlands). The expression cassettes containing bagworm silk-protein motif sequence were transformed into an *A. oryzae* protease-deficient strain (M5063) and a ∆*creA* strain (M6752) through protoplast-mediated transformation and *pyrG*-dependent screening as described above. The transformants were confirmed by PCR using primers toward the target genes and loci to confirm the correct integration (Table S1). Spore suspensions of the correct transformants were prepared by growing the fungus on potato dextrose agar plates for 5 d. Spores were harvested and suspended in a spore suspension buffer (0.8% NaCl, 0.025% Tween 20, and 20% glycerol) through cotton and stored at −80 °C prior further analysis.

### Small-scale 24-well plate cultivation

To screen for the production of bagworm silk-inspired proteins, 24-well plate cultivations were performed. Ten microliters of spore suspension was inoculated in 3 ml of buffered glucose-complex medium (BMGY: 1% yeast extract, 2% peptone, 100 mM potassium phosphate pH 6.0, 1.34% yeast nitrogen base with ammonium sulfate, 4 × 10^−5^% biotin, and 2% glucose) at 28 °C, 800 rpm, and 80% humidity for 5 d with or without protease inhibitor supplementation. For protease inhibitor supplementation, 10 μM chymostatin, 10 μM pepstatin, and 0.5 mg/ml soybean trypsin inhibitor (final concentration) were added to the cultures on days 2, 3, and 4 after inoculation. After 5 d, the supernatant was collected by centrifugation at 3,220 × *g* for 10 min and stored at −20 °C prior to further analysis.

### Two-liter bioreactor cultivation

The preculture was prepared by inoculation of 1 × 10^7^ spores into 50 ml of BMGY with 0.2% agar and incubating at 30 °C and 200 rpm until a small pellet was formed, for 8 h (wild type) or 16 h (H-eBGW). The cultivation medium contained 10 g/l glucose, 5 g/l yeast extract, 15 g/l KH_2_PO_4_, 0.52 g/l KCl, 0.54 g/l MgSO_4_, and 1 ml/l Hutner’s trace elements solution. Protease inhibitors, as mentioned in the previous section, were added on days 2 and 3. Foaming was controlled with the antifoam agent Struktol J673A. Parallel fed-batch cultivations were carried out in 2-l bioreactors (Biostat CT2, Braun Biotech International, Germany) equipped with Rushton impellers with a 5.3-cm diameter for agitation and a ring sparger for aeration. The initial volume was 1.5 l with a 2% inoculum; reactors were fed 300 g/l glucose and operated for 96 h at 28 °C, pH 6, and 1 l/min aeration. For the wild type, the initial feed rate was 1.8 g glucose/h and was lowered to 1.2 g/h at around 60 h. For H-eBGW, the initial feed rate was 1.9 g glucose/h. During cultivation, the feed rate was manually adjusted based on CO_2_ data to prevent glucose accumulation, reaching a final rate of 1.35 ± 0.15 g/h. The pH was adjusted using 15% H_3_PO_4_ and 12.5% NH_4_OH; 50 ml of sterile water was added to both bioreactors on days 2 and 3 to reduce the viscosity of the filamentous growth, which could damage the impellers. The final volume was 1.8 and 1.85 l for the wild type and H-eBGW, respectively. To collect the supernatant, the culture was filtered through XE50H and XE1200H filter sheets, respectively (FILTROX Carlson Ltd., Barnoldswick, United Kingdom) followed by filtration through a 0.45-μm PES membrane (Nalgene Rapid-Flow Sterile Disposable Bottle Top Filter, Thermo Fisher Scientific, Nuevo León, Mexico). The cell dry weight was measured by vacuum filtration of the sample on Whatman GF/B glass fiber filter paper, and the washed biomass was dried at 105 °C overnight. The supernatant was collected from filtration and stored at −20 °C prior to further analysis.

### Protein purification

After the filtration process, the supernatant was concentrated with buffer exchange to PBS pH 7.3 using Vivaflow 200 and a 10-kDa-MWCO PES membrane (Sartorius Stedim Lab Ltd., Stonehouse, United Kingdom). For C-tag purification, the concentrated supernatant was filtered (Whatman FP 30/0.2 CA-S, 0.22 μm; Cytiva, Sweden) and applied to 1 ml of CaptureSelect C-tagXL Pre-packed Column (Thermo Scientific, Leiden, the Netherlands) equilibrated with PBS pH 7.3. The protein was eluted by 20 mM Tris–HCl, 2 M MgCl_2_, and 1 mM EDTA pH 7.5 (10 column volume, flow rate 1.0 ml/min). The column was regenerated using 0.1 M glycine pH 2.7. For gel filtration, the concentrated supernatant was filtered (0.22 μm) and desalted onto prepacked PD MidiTrap G-25 (Cytiva) equilibrated with 50 mM MES (pH 6.5, 8.3 ml) loaded with 2.5 ml of the concentrated sample (containing 38 mg total protein/ml), and 3.5 ml was collected (*A*_280_ = 21.13). The collected protein fraction was then applied to a prepacked HiLoad 26/60 Superdex 75 pg (Pharmacia Biotech, Sweden) equilibrated with 25 mM MES pH 6.5 and 0.2 M NaCl (flow rate: 1.0 ml/min). Fractions containing bagworm silk protein were pooled together based on the Western blot results, and part of this pool was 6× concentrated using 5-kDa-MWCO Vivaspin and repurified on prepacked HiLoad 26/60 Superdex 75 pg (Pharmacia Biotech, Sweden) as mentioned above. All purification steps were performed using an ÄKTA-Pure FPLC system (GE Healthcare, Sweden). The purified samples were stored at −20 °C and further used in spin-coating for nanomechanical tests.

### Protein analysis

SDS-PAGE was performed using 4% to 20% Criterion TGX Precast Midi Protein Gel (Bio-Rad, Hercules, CA) and Prestained Protein Standards (Precision Plus Protein All Blue, Bio-Rad). Coomassie blue staining was performed using PageBlue Protein Staining Solution (Thermo Scientific, Rockford, IL). Western blotting was performed using the Trans-Blot Turbo Transfer System (Bio-Rad). After blocking the membrane using TBS-T (100 mM Tris–HCl, 150 mM NaCl, and 0.05% Tween 20) containing 5% w/v skimmed milk powder, C-tagged proteins were detected using CaptureSelect Biotin Anti-C-tag Conjugate (1:2,000 v/v, Thermo Fisher Scientific, Life Technologies Europe BV, Bleiswijk, the Netherlands) followed by IRDye 800CW Streptavidin (1:5,000 v/v, LI-COR Biosciences, Lincoln, NE), and the signal was detected using the Odyssey CLx Imaging System Unit (LI-COR Biosciences). Deglycosylation was performed by using endoglycosidase H (New England Biolabs) as recommended by the manufacturer. The total protein was analyzed using the DC Protein Assay Kit (Bio-Rad).

### Spin-coating of thin films for nanomechanical testing and WCA

Samples containing bagworm silk-inspired protein were thawed on ice, mixed thoroughly, and centrifugated at 10,000 rpm for 10 min; the supernatant was taken for spin-coating. Prior to spin-coating, silica wafers (1 × 1 cm^2^; Compugraphics, Jena, Germany) were placed in an ultraviolet ozonizer (UV/OzoneProCleaner, BioForce Nanosciences, Ames, IA) for 15 min and then wetted with pure toluene, followed by spin-coating of the toluene solution. An 80-μl aliquot of the H-eBGW-containing pooled fraction, with a total protein concentration of 1 to 34 g/l, was added on the top of silica and allowed to stand at ambient temperature for 10 min prior to spinning. The protein concentration used for spin-coating refers to the total protein concentration of the pooled fraction used for film preparation. The spin-coating was performed in a Autolab spin-coater device (Ekochemie, Germany) at 2,000 rpm for 2 min. Spin-coated films were treated at different temperatures (22, 80, and 140 °C) for 10 min in the oven [75,76].

### Contact angle measurement of wettability

The wettability of the thin films was determined by static WCA measurements using the sessile drop method in a CAM 200 goniometer (KSV Instruments, Finland) equipped with a video camera at room temperature. The WCA was measured from the drop shape using the Young–Laplace equation. The WCA was recorded (3 times per second) within 60 s immediately after placing the droplet (2 μl) on the surface of the spin-coated film; the WCA was calculated as the average value of the measurements.

### Nanomechanical testing

Nanomechanical tests were carried out using an iNano nanoindenter (KLA Corp., USA) with a Berkovich diamond tip (Synton-MDP, Switzerland) equipped with an InForce 50-mN electromagnetic force actuator with a 100-kHz data acquisition rate and a 20-μs time constant, a dynamic indentation module (continuous stiffness measurement [CSM]), a NanoBlitz module for high-speed nanoindentation for 2-dimensional (2D) large-area mapping, and a scratch test module (continuous stiffness measurement [CSM]), NanoBlitz for high-speed nanoindentation to creating 2-dimensional (2D) large area mapping module, and a scratch test module. The Oliver and Pharr method was used to calibrate the diamond area function of the tip as described previously [75,76]. For the CSM measurements, a target load of 50 mN, an indentation strain rate of 0.200 s^−1^, a frequency of 110 Hz, and a target displacement of 2 nm were used. For the 2D mapping, an area of 100 × 100 μm was selected. For the scratch test, we performed the measurement using a scratch length of 150 μm and with a scratch starting load of 0.04 mN and a maximum load of 20 mN using a 20 μm/s scratch velocity.

## Conclusion

In the quest for materials that couple technological ambition and environmental responsibility, we demonstrate the possibility of producing bagworm silk-inspired protein variants using a protease-deficient *A. oryzae* strain. Even though the fraction containing bagworm silk-inspired-protein (H-eBGW) showed promising coating properties, the target proteins could be produced in a 2-l bioreactor only when supplemented with protease inhibitors. This suggests that additional proteases capable of degrading the silk variants remain active in the production strains that should be identified and removed. Moreover, the detected predominant background proteins, amylases, make their genomic loci suitable targets for integration of additional silk expression cassettes to increase yield while simultaneously simplifying downstream processing. Ongoing strain engineering efforts aim to generate a production host better suited for silk-based protein production, creating new opportunities for designing next-generation materials. Our bagworm silk-inspired-protein-containing fractions could be processed into functional coatings whose surface wetting behavior is adjustable by a simple posttreatment step. The expression of this heterologous silk-inspired protein is a promising showcase for the future development of innovative materials and reshapes our relationship with the material world.

## Data Availability

Data will be made available upon reasonable request. The plasmids are available upon request under a material transfer agreement.
