# Supplementary material for: Design and Heterologous Production of Bagworm Silk-Inspired Proteins in Aspergillus oryzae
Source: Comput Struct Biotechnol J. 2026 Sep 30;35(1):0233. doi: 10.34133/csbj.0233 (PMC13624362; doi:10.34133/csbj.0233)
Supplement: Supplementary file 1 [file csbj.0233.f1.docx]

**Supplementary Materials**

**Design and heterologous production of bagworm silk-inspired proteins in *Aspergillus oryzae***

Adiphol Dilokpimol*, Ulrike Abendroth, Mari Nyyssönen, Anna S. Borisova, Anni Seisto, Jenni Muli, Dominik Mojzita, Fiona Småros, Hans Mattila, Antti P. Aalto, Mari Valkonen, Pezhman Mohammadi*

**Affiliation:**

VTT Technical Research Center of Finland, Maarintie 3, 02150 Espoo, Finland

**Corresponding author email address**

adiphol.dilokpimol@vtt.fi, pezhman.mohammadi@vtt.fi

**The construction of the *A. oryzae* strain** **M5063**

The *A. oryzae* strain (NRRL 5590 / ATCC 42149 / RIB 40) was used as a starting background strain, and multiple construction steps were performed to delete selected genes and to integrate synthetic transcription factors (sTF) gene – to create a basic production strain with low protease activity and ready for SES-based expression of target genes. Here, the description of genetic modification steps: **1)** A co-transformation of *pyrG* and *ligD* deletion cassettes, both with the sTF expression cassettes. The idea was to obtain a strain where the *pyrG* gene would be replaced by the sTF cassette, and simultaneously the *ligD* gene would be also replaced by the sTF cassette. The deletion of *pyrG* would be then used for subsequent constructions (using *pyrG* as a selection marker), and the deletion of *ligD* would enhance the homologous recombination (HR). The sTF cassette would be providing the sTF for later expression of target genes controlled by the SES-promoter system. The transformants were selected on the 5-FOA-containing agar. Several colonies, which grew in presence of 5-FOA, were tested, but no intended strain was found. However, one colony was obtained, where both *pyrG* and *ligD* were deleted, but no sTF cassette was present. We decided to carry on with that strain to proceed with other deletions. **2)** Deletion of *ptrT* gene was achieved by transformation of the *ptrT* - deletion cassette with *pyrG* marker gene with a loop-out sequence, the transformants were obtained on agar without uracil/uridine. The idea was to remove the *pyrG* cassette by HR and select for such strain in presence of 5-FOA in subsequent cultivation. However, *A. oryzae* is very tolerant for 5-FOA and even in high 5-FOA conditions (2g/L) some colonies contained *pyrG* marker still grew, in addition the cultivations were relatively long (slow growth). We decided instead, to facilitate the *pyrG* loop-out by secondary transformation with “empty” cassette. This approach was successful and much faster; therefore, it was employed for other construction steps too. **3)** Replacement of *alpA* gene by the sTF cassette was done by transformation of the construct containing *pyrG* marker and the sTF expression cassette. The correct clone was subsequently transformed by the “pyrG-loopout” cassette, which contained only the sTF cassette without pyrG marker. **4)** Deletion of *ocpB* gene was achieved by transformation of the pyrG-containing *ocpB* deletion cassette, followed by secondary transformation with pyrG-less *ocpB* cassette. **5)** Deletion of *cpyA* gene was achieved by transformation of the pyrG-containing *cpyA* deletion cassette, followed by secondary transformation with pyrG-less *cpyA* cassette. **6)** Deletion of *Np1* gene was achieved by transformation of the pyrG-containing *Np1* deletion cassette. The correct clone was subsequently transformed by the “pyrG-loopout” cassette introducing a second sTF expression cassette, essentially replacing the pyrG marker with the sTF cassette in the strain. **7)** The final deletion of *tppA* gene was achieved by transformation of the pyrG-containing *tppA* deletion cassette, followed by secondary transformation with pyrG-less *tppA* cassette. The final strain (M5063) contains the following deletions/modifications: ∆*ligD*; ∆*ptrT*; ∆*alpA* (+sTF); ∆*ocpB*; ∆*cpyA*; ∆*Np1* (+sTF); ∆*tppA*; and ∆*pyrG*.

**Figure S1 Plasmids used in this research**

Maps of the deletion and expression DNA-cassettes. The synthetic transcription factor (sTF) encoding the fusion construct composed of a DNA-binding protein (Bm3R1) and transcription activation domain (AD, expressed from the An_00850cp), and the target-gene expression cassette contain 8x binding sites for Bm31R (8xBS) and An_201cp – forming the target gene promoter – are from [1]. eBGW and eBGW1 were codon optimized for *A. oryzae*.

1. *ligD*-deletion

2. *pyrG*-deletion

3. *creA*-deletion

4. *ptrT*-deletion (and pyrG-“loop-out”-cassette)

5. *alpA*-deletion + sTF insertion (and pyrG-“loop-out”-cassette)

6. *ocpB*-deletion (and pyrG-“loop-out”-cassette)

7. *cpyA*-deletion (and pyrG-“loop-out”-cassette)

8. *Np1*-deletion (and pyrG-“loop-out” + sTF insertion cassette)

9. *tppA*-deletion (and pyrG-“loop-out”-cassette)

10. AmyAss-N28-eBGW-C-tag

11. HFBss-eBGW-C-tag

12. HFBss-N28-eBGW-C-tag

13. AmyAss-N28-eBGW1-C-tag

14. HFBss-eBGW1-C-tag

15. HFBss-N28-eBGW1-C-tag

16. AmyAss-N28-eBGW-C-tag

17. HFBss-eBGW-C-tag

18. HFBss-N28-eBGW-C-tag

19. AmyAss-N28-eBGW1-C-tag

20. HFBss-eBGW-C-tag

21. HFBss-N28-eBGW-C-tag

**Figure S2**. **Production of two silk variants in *A. oryzae* protease-deficient strain (M5063).**

Culture broth (11.25 µL supernatant) from different constructs (5 days of cultivation in BMGY, 28⁰C, 24-well plate) were screened for silk variant production. **a)** Western blot analysis using an anti C-tag antibody. **b)** Coomassie blue stained SDS-PAGE. M, protein marker and C, *A. oryzae* protease-deficient strain without silk variant as a negative control.

**Figure S3. Estimated concentration of e-BGW and e-BGW1 by densitometric analysis of Figure 4.**

The e-BGW and e-BGW1 concentrations were estimated based on the signal was detected using Odyssey CLx Imaging System Unit and Image Studion v 6.0 (LI-COR Biosciences).

**Figure S4. Deglycosylation of silk variants by endoglycosidase H or Peptide:N-glycosidase F treatment**

The culture broth with silk variants from 5 days of cultivation in 24-well plate with protease inhibitors supplementation were treated with endoglycosidase H (Endo H), (**a, b**) and the supernatant containing H-eBGW from 5 days of cultivation in 2-L bioreactor were treated with Peptide:N-glycosidase F (PNGase F), (**c, d**). **a)** Western blot analysis using an anti C-tag antibody. **b)** Coomassie blue staining. **c)** Western blot analysis using an anti C-tag antibody. **d)** Coomassie blue staining. M; protein marker, + H; treated with Endo H, + F; treated with PNGase F, –; not treated with Endo H or PNGaseF.

**Figure S5. Biomass accumulation of 2-L bioreactor cultivation of H-eBGW and wild type strains.**

The cell dry weight (CDW) was measured by vacuum filtration of 5 mL culture broth on Whatman GF/B glass fibre filter paper, and washed biomass was dried at 105⁰C overnight.

**Figure S6. Purification of H-eBGW by C-tag affinity chromatography.**

The filtered culture broth after harvesting was first concentrated and buffer exchanged by ultrafiltration and subsequently purified by C-tag using CaptureSelect TM C-tagXL pre-packed column. **a)** Western blot analysis using an anti C-tag antibody. **b)** Coomassie blue staining. M; protein marker, FB; filtered culture broth after harvesting, FT1-3; flow through during ultrafiltration at different time points, C; concentrated broth (10 time diluted) after ultrafiltration, FT; flow through from C-tag purification, F2-5; purified fractions. Amy indicates amylases and SBTI indicates soybean trypsin inhibitor.

**Table S1 List of primers used in this research**

| **Primer name** | **Sequence** | **Primer function** |
| --- | --- | --- |
| dligDF | GTTTATCGCAGGTTTGAAATCC | Check deletion of *ligD* |
| dligDR | GACGACATCCAACGACTCTC | Check deletion of *ligD* |
| dpyrGF | TGACAACCACCAAAGAGCTG | Check deletion of *pyrG* |
| dpyrGR | GATGAGGAAATTGTGCTTCTCTG | Check deletion of *pyrG* |
| dcreAF | CATACGGGTGAAAAGCCACA | Check deletion of *creA* |
| dcreAR | AGTTTACGCTGTGTTCCGTC | Check deletion of *creA* |
| dptrTF | CGTCCATTCCATTTACCACAG | Check deletion of *ptrT* |
| dptrTR | CCCATTCAACTTTCCATCGT | Check deletion of *ptrT* |
| dalpAF | GAGCACCGACTACATCTACGA | Check deletion of *alpA* |
| dalpAR | GCGATACCATAAGTCTTGCCA | Check deletion of *alpA* |
| docpBF | CCATCTTTCACTGCTTTCTTCC | Check deletion of *ocpB* |
| docpBR | CCTCCATCGCCATATCATATACAG | Check deletion of *ocpB* |
| dcpyAF | GTCCCTGCCTCGATTTACTG | Check deletion of *cpyA* |
| dcpyAR | CATAACCACCAACTTCGGCTC | Check deletion of *cpyA* |
| dnp1F | CGTGCTTACCCATTCTCAACC | Check deletion of *Np1* |
| dnp1R | CGAGATACTTGCCGTCAGTC | Check deletion of *Np1* |
| dtppAF | AGCCTCTTTATGTCCCTTACC | Check deletion of *tppA* |
| dtppAR | TATTGTCAGCACCATAATCCAG | Check deletion of *tppA* |
| sTF_F | GTCACATTCACAAAGAACCATCC | Check insertion of sTF (Bm3R1-AD) |
| sTF_R | GACCTCCATAAACGATCCGA | Check insertion of sTF (Bm3R1-AD) |
| Amy5'F | ATTGGATATGACAATGGAATCGTG | Check integration of *amyC* 5’ |
| Amy5'R | GACGCCATCTTGATGCTTC | Check integration of *amyC* 5’ |
| Amy3'F | CAATGTCGAGCACGGGTAGT | Check integration of *amyC* 3’ |
| Amy3'R | TGACTTATTGGACCTTTTGGAG | Check integration of *amyC* 3’ |
| GaaC5'F | GACTGAGTATCCACGAGTTTTG | Check integration of *gaaC* 5’ |
| GaaC5'R | GACGCCATCTTGATGCTTCTTTTC | Check integration of *gaaC* 5’ |
| GaaC3'F | CTGGCCCTCTTCATCTTCCA | Check integration of *gaaC* 3’ |
| GaaC3'R | CCTCCGAGCAGTTGGAAG | Check integration of *gaaC* 3’ |
| BM3R1F | GCTAGTTCTCCCCGGAAACT | Check the presence of eBGW/eBGW1 |
| TglaAR | CGGAGGAGCCTTACATGGTC | Check the presence of eBGW/eBGW1 |

**Reference**

1. Rantasalo A, Landowski CP, Kuivanen J, Korppoo A, Reuter L, Koivistoinen O, Valkonen M, Penttilä M, Jäntti J, Mojzita D. A universal gene expression system for fungi. *Nucleic Acids Research* 2018; 46(18):e111–e111.
